# Quality of Voluntary Medical Male Circumcision Services during Scale-Up: A Comparative Process Evaluation in Kenya, South Africa, Tanzania and Zimbabwe

**DOI:** 10.1371/journal.pone.0079524

**Published:** 2014-05-06

**Authors:** Larissa Jennings, Jane Bertrand, Dino Rech, Steven A. Harvey, Karin Hatzold, Christopher A. Samkange, Dickens S. Omondi Aduda, Bennett Fimbo, Peter Cherutich, Linnea Perry, Delivette Castor, Emmanuel Njeuhmeli

**Affiliations:** 1 Johns Hopkins Bloomberg School of Public Health, Department of International Health, Baltimore, Maryland, United States of America; 2 Tulane University School of Public Health and Tropical Medicine, Department of Global Health Systems and Development, New Orleans, Louisiana, United States of America; 3 The Centre for HIV and AIDS Prevention Studies, Johannesburg, South Africa; 4 Population Services International, Harare, Zimbabwe; 5 Institute of Continuing Health Education, University of Zimbabwe, College of Health Sciences, Harare, Zimbabwe; 6 Impact Research and Development Organization, Kisumu, Kenya; 7 Ministry of Health and Social Welfare, National AIDS Control Programme, Dar es Salaam, Tanzania; 8 National AIDS and Sexually Transmitted Infections Control Program, Nairobi, Kenya; 9 United States Agency for International Development, Washington, D.C., United States of America; World Health Organization, Switzerland

## Abstract

**Background:**

The rapid expansion of voluntary medical male circumcision (VMMC) has raised concerns whether health systems can deliver and sustain VMMC according to minimum quality criteria.

**Methods and Findings:**

A comparative process evaluation was used to examine data from SYMMACS, the Systematic Monitoring of the Voluntary Medical Male Circumcision Scale-Up, among health facilities providing VMMC across two years of program scale-up. Site-level assessments examined the availability of guidelines, supplies and equipment, infection control, and continuity of care services. Direct observation of VMMC surgeries were used to assess care quality. Two sample tests of proportions and t-tests were used to examine differences in the percent of facilities meeting requisite preparedness standards and the mean number of directly-observed surgical tasks performed correctly. Results showed that safe, high quality VMMC can be implemented and sustained at-scale, although substantial variability was observed over time. In some settings, facility preparedness and VMMC service quality improved as the number of VMMC facilities increased. Yet, lapses in high performance and expansion of considerably deficient services were also observed. Surgical tasks had the highest quality scores, with lower performance levels in infection control, pre-operative examinations, and post-operative patient monitoring and counseling. The range of scale-up models used across countries additionally underscored the complexity of delivering high quality VMMC.

**Conclusions:**

Greater efforts are needed to integrate VMMC scale-up and quality improvement processes in sub-Saharan African settings. Monitoring of service quality, not just adverse events reporting, will be essential in realizing the full health impact of VMMC for HIV prevention.

## Introduction

Given the scientific evidence of the benefits of male circumcision in reducing HIV incidence rates among men [1]–[3], several sub-Saharan African countries have begun to scale-up voluntary medical male circumcision (VMMC) services for adult and adolescent males [4]–[6]. High coverage of safe VMMC services could prevent up to 3.4 million new HIV infections [7]. However, the rapid expansion of VMMC programs has raised questions regarding the extent to which health systems can deliver VMMC according to minimum quality standards which are sustained at-scale [8], [9]. Ensuring correct performance of VMMC procedures during scale-up is essential in realizing the potential health impact of averted HIV infections and in reducing health systems costs for HIV-related care [10], [11].

In 2009, the World Health Organization (WHO) developed a quality assessment toolkit to assess a range of quality standards relating to facilities' VMMC supply inventory, provider competence, surgical technique, infection prevention, and continuity of care [12]. Preliminary findings suggest that VMMC surgical complications have resulted from inadequate provider competence and training, poor clinical infrastructure, and limited patient information and follow-up [2], [5]. Yet, there is a dearth of published data on other aspects of service quality among VMMC programs in resource-poor settings [5], [13], [14]. Most quality assessments have focused on occurrence of adverse events [8], [14]–[16], device malfunction rates [10], procedure duration [10], or patient acceptability and resumption of sexual activity [13]. Less attention has been given to the availability of minimum service components and provider performance of specific surgical tasks in contexts where countries are rapidly expanding the number of VMMC sites [2], [13], [14]. To our knowledge, this study is the first published to-date to address this gap by examining quality of VMMC services among four African countries over two years of program scale-up.

## Methods

### Study Design

A comparative process evaluation was used to examine VMMC service provision over time. Data were drawn from SYMMACS, the Systematic Monitoring of the Voluntary Medical Male Circumcision Scale-Up, which assessed the evolution of VMMC scale-up in Kenya, South Africa, Tanzania, and Zimbabwe. SYMMACS conducted a two-stage multi-country evaluation with two cross-sectional data collection periods (one in 2011 and one in 2012) among health facilities providing VMMC and selected VMMC surgeries. A detailed description of the SYMMACS evaluation and sampling methodology is provided in this supplement [17].

### Scale-up Setting

The scale-up model used to expand the number of VMMC sites varied by country. Kenya and Tanzania adopted a focused implementation strategy using outreach sites and fixed clinical facilities supported by multiple implementing agencies within regions with the highest HIV prevalence and lowest male circumcision rates. Kenya additionally provided services via mobile sites to deliver VMMC in underserved areas. South Africa adopted a national approach using outreach and fixed sites with multiple partners, while Zimbabwe's national program was implemented by public sector service providers in outreach and fixed sites supported by a single implementing partner. Kenya was the only country with a fully-scaled VMMC program in the evaluation's first and second years, while Tanzania, South Africa, and Zimbabwe substantially increased the number of VMMC sites from two- to five-fold over the evaluation period.

### Measurement

The comparative process evaluation measured two outcomes: VMMC facility preparedness and quality of VMMC surgeries. *VMMC facility preparedness* was assessed by direct observation using a pre-tested observation checklist based on the WHO VMMC quality assessment tool [12]. The checklist included items grouped into five preparedness categories: “guidelines and protocols” comprised 3 standard operating protocols; “equipment and supplies” included 9 items relating to anesthesia, antibiotics, condoms, and other supplies; “basic life support” (BLS) consisted of 4 items: cardiopulmonary resuscitation bag and mask, oxygen, intravenous lines, and anaphylaxis medications; “hygienic and safe infrastructure” consisted of 4 items measuring adequacy of lighting, ventilation, cleanliness, and adverse event monitoring; and “patient services” included 5 items: circumcision, group education, individual counseling, HIV counseling and testing, and referral services. *Quality of VMMC surgeries* was evaluated by direct observation of randomly selected surgeries using a checklist of tasks across three surgical periods: pre-operative (1 item), operative (21 items), and post-operative (7 items). Example tasks included pre-operative examination, use of sterile processes, correct surgical techniques, and provision of post-operative monitoring and counseling. Operative period tasks were further separated into two categories: ‘safe operative procedures’ (8 items) relating to surgical safety and ‘operative techniques’ (13 items) relating to step-by-step circumcision procedures. Trained observers scored items as “unsatisfactory” (scored = 0), “partially satisfactory” (scored = 1), and “satisfactory” (scored = 2).

### Statistical Analysis

Data were analyzed using STATA (Version 12). Two sample tests of proportions and t-tests were used to examine differences in facility preparedness and VMMC service quality by country between 2011 and 2012. Composite measures of facility preparedness and service quality were calculated based on the percent of facilities with all requisite items per category and the mean number and mean percent of items scored as “satisfactory” per category. Item-level differences between time points were also assessed using Pearson's chi-squared tests. A significance level of *p*<.05 was used in all analyses.

### Ethical Considerations

Ethical approval was obtained from the Tulane University Institutional Review Board (United States), the Kenya Medical Research Institute, the University of Witwatersrand's Human Research Ethics Committee (South Africa), the Tanzanian National Institute for Medical Research, and the Medical Research Council of Zimbabwe.

## Results

### VMMC Facility Preparedness

Table 1 presents changes in the percent of facilities having all preparedness items per category. During the initial year, facilities in all four countries had an average of 1.3 to 2.0 guidelines on-site at the time of assessment. Across countries, less than half of facilities (Kenya: 34.5%, South Africa: 33.3%, Tanzania: 7.7%, Zimbabwe: 7.1%) had all three guidelines. In the second year, the percent of facilities having all three guidelines significantly improved in Zimbabwe (+34.6, 95% CI: 10.6, 58.4). However, no significant changes occurred in the remaining countries: Kenya (+19.1, 95% CI: −6.2, 44.4), Tanzania (+19.9, 95% CI: −1.9, 41.7), South Africa (−20.8, 95% CI: −46.8, 5.1).

**Table 1:** Summary of Availability of VMMC Protocols, Equipment, Supplies, and Services from Initial Year (2011) to Expansion Year (2012).

	Kenya			South Africa			Tanzania			Zimbabwe		
	2011 (T_0_)	2012 (T_1_)	Δ = T_1_- T_0_	2011 (T_0_)	2012 (T_1_)	Δ = T_1_- T_0_	2011 (T_0_)	2012 (T_1_)	Δ = T_1_- T_0_	2011 (T_0_)	2012 (T_1_)	Δ = T_1_- T_0_
Number of facilities	30	29		15	40		14	29		14	24	
**Guidelines**	n = 28	n = 29		n = 15	n = 40		n = 13	n = 29		n = 14	n = 24	
Percent (%) facilities with all 3 guidelines	34.5	53.6	+19.1	33.3	12.5	−20.8	7.7	27.6	+19.9	7.1	41.7	**+34.6** *
Mean number (%) guidelines^A^	1.8 (60%)	2.0 (68%)	+0.2 (+8%)	2.0 (67%)	1.0 (35%)	**−1.0** * **(−32%** * **)**	1.6 (54%)	1.8 (62%)	+0.2 (+8%)	1.3 (43%)	2.1 (69%)	**+0.8** * **(+26%** * **)**
**Equipment/Supplies**	n = 28	n = 28		n = 15	n = 39		n = 13	n = 29		n = 14	n = 24	
Percent (%) facilities with all 9 equipment and supplies	25.0	10.7	−14.3	50.0	2.6	**−47.4** *	23.1	6.9	−16.2	28.6	45.8	+17.2
Mean number (%) equipment and supplies items^A^	7.8 (87%)	7.5 (83%)	−0.3 (−4%)	8.3 (92%)	6.6 (73%)	**−1.7** * **(−19%** *	8.2 (91%)	7.7 (86%)	**−0.5** * **(−5%** * **)**	7.9 (88%)	8.4 (94%)	**+0.5** * **(+6%** * **)**
**Basic Life Support**	n = 29	n = 29		n = 15	n = 40		n = 12	n = 29		n = 14	n = 24	
Percent (%) facilities with all 4 basic life support items	0	3.4	+3.4	64.3	27.5	**−36.8** *	7.7	17.2	+9.5	35.7	58.3	+22.6
Mean number (%) basic life support items^A^	0.8 (20%)	1.5 (37%)	**+0.7** * **(+17%** * **)**	3.2 (80%)	2.3 (57%)	**−0.9** * **(−23%** * **)**	1.5 (37%)	2.1 (53%)	+0.6 (+16%)	2.3 (57%)	2.8 (70%)	+0.5 (+13%)
**Hygiene and Safety**	n = 29	n = 29		n = 15	n = 40		n = 12	n = 29		n = 14	n = 24	
Percent (%) facilities with all 4 hygiene and safety items	75.9	86.2	+10.3	53.3	12.5	**−40.8** *	33.3	17.2	−16.1	28.6	83.3	**+54.7** *
Mean number (%) hygiene and safety items^A^	3.6 (91%)	3.8 (95%)	+0.2 (+4%)	3.2 (82%)	2.4 (61%)	**−0.8** * **(−21%** * **)**	3.0 (75%)	2.9 (73%)	−0.1 (−2%)	2.3 (57%)	3.6 (89%)	**+1.3** * **(+32%** * **)**
**Service Package**	n = 28	n = 29		n = 15	n = 40		n = 14	n = 29		n = 14	n = 24	
Percent (%) facilities with all 5 package services	32.1	58.6	**+26.5** *	86.7	22.5	**−64.2** *	85.7	58.6	−27.1	85.7	100	+14.3
Mean number (%) package services^A^	4.3 (86%)	4.5 (90%)	+0.2 (+4%)	4.8 (96%)	3.5 (70%)	**−1.3** * **(−26%** * **)**	4.9 (98%)	4.6 (92%)	−0.3 (−6%)	4.9 (98%)	5.0 (100%)	+0.1 (+2%)

[A] Includes only percent of facilities with total score ( = 1 point). Items receiving a partial score ( = 0.5 point) at the facility were not counted as available.

* Denotes statistically significant at p<0.05.

Health facilities in all four countries reported having most of the 9 listed equipment and supplies during the initial year (Kenya: 7.8, South Africa 8.3, Tanzania 8.2, Zimbabwe 7.9). With the exception of South Africa, roughly a quarter of facilities had all 9 items (Kenya 25%, South Africa 50%, Tanzania 23%, Zimbabwe 29%). As compared to the expansion year, availability of supplies and equipment significantly declined in South Africa from 50.0% of facilities having all 9 items to 2.6% of facilities (−47.4, 95% CI: −74.1, −20.8). No statistically significant changes were observed in the other countries, although the general trend was a decline in supply availability: Kenya (−14.3, 95% CI: −34.0, 5.4), Tanzania (−16.2, 95% CI: −40.9, 8.9), and Zimbabwe (+17.2, 95% CI: −13.7, 48.2). Sites in Kenya and Tanzania scored poorly on available BLS equipment, ranging from an average of 0.8 to 1.5 of the 4 recommended items. These levels increased slightly, but remained low during the expansion year, with 3.4% and 17.2% of facilities having all 4 BLS items. Although more facilities in South Africa (27.5%) had all 4 BLS items, this represented a significant decline from 64.3% of facilities in its initial year (−36.8, 95% CI:−65.4, −8.1). In Zimbabwe, approximately half (58.3%) of facilities had all 4 BLS items during the second year compared to 35.7% during the initial year (+22.6, 95% CI: −9.3, 54.5).

Similar patterns were observed for hygienic and safe infrastructure. Kenya maintained relatively high scores with no significant changes with 75.9% and 86.2% of facilities having all 4 items each year, respectively (+10.3, 95% CI: −9.7, 30.3). Tanzania maintained relatively low scores with 33.3% and 17.2% of facilities (−16.1, 95% CI: −46.1, 13.9). Higher scores in South Africa significantly declined from 53.3% to 12.5% of facilities (−40.8, 95% CI: −68.1, −13.6). Lower scores in Zimbabwe significantly improved from 28.6% to 83.3% (+54.7, 95% CI: 26.8, 82.8). Most facilities in Kenya, Tanzania, and Zimbabwe had high service availability, averaging 4.3 to 4.9 of the 5 package services in 2011, which were maintained during expansion. Significant service erosion was observed in South Africa from 4.8 to 3.5 of the 5 package services.

Table 2 shows item-level analyses of facility preparedness. Protocol availability varied among countries. Supplies most commonly unavailable were HIV post-exposure prophylaxis, followed by antibiotics and condoms. Absence of oxygen and anaphylaxis medications appeared to drive low BLS scores, while absence of adverse event monitoring systems and lighting drove lower infrastructure scores. With the exception of referral services, most VMMC facilities offered group education, individual counseling, and HIV counseling and testing.

**Table 2:** Item-Level Availability of VMMC Protocols, Equipment, Supplies, and Services from Initial Year (2011) to Expansion Year (2012).

*Indicator:* Percent (%) of facilities with Total Score ( = 1 point) by Item^A^												
	Kenya			South Africa			Tanzania			Zimbabwe		
	2011 (T_0_)	2012 (T_1_)	Δ = T_1_- T_0_	2011 (T_0_)	2012 (T_1_)	Δ = T_1_- T_0_	2011 (T_0_)	2012 (T_1_)	Δ = T_1_- T_0_	2011 (T_0_)	2012 (T_1_)	Δ = T_1_- T_0_
Number of facilities	30	29		15	40		14	29		14	24	
**Guidelines and Protocols** *(3 items)*												
VMMC Standard Operating Procedures (SOP)	70.0	79.3	-	40.0	42.5	-	50.0	86.2	**+36.2** *	21.4	45.8	-
Post-Exposure (PE) Prophylaxis Guidelines	56.7	65.5	-	86.7	32.5	**−54.2** *	21.4	62.1	**+40.7** *	50.0	70.8	-
National Protocols for Syndromic Management and Treatment of Sexually Transmitted Infections (STI)	46.7	55.2	-	73.3	30.0	**−43.3** *	85.7	37.9	**−47.8** *	57.1	91.7	**+34.6** *
**Equipment and Supplies** *(9 items)*												
Sterilized VMMC Instruments	100	100	-	86.7	95.0	**-**	92.9	100	-	92.9	100	-
Local Anesthesia	93.3	96.6	-	100	90.0	**-**	92.9	100	-	100	100	-
Antibiotics	76.7	58.6	-	80.0	60.0	**-**	78.6	89.7	-	85.7	95.8	-
Pain Medication	93.3	100	-	100	75.0	**-**	92.9	100	-	100	100	-
Antiseptic Solution	100	100	-	100	87.5	**-**	92.9	100	-	85.7	100	-
Dressing Materials	100	100	-	100	77.5	**-**	92.9	100	-	100	100	-
HIV Post-Exposure Prophylaxis	46.7	17.2	**−29.5** *	60.0	7.5	**−52.5** *	57.1	10.3	**−46.8** *	57.1	50.0	-
Sharps Container	93.3	100	-	100	95.0	-	92.9	100	-	100	100	-
Distributable Male Condoms	70.0	72.4	-	86.7	70.0	-	78.6	72.4	-	71.4	100	**+28.6** *
**Basic Life Support** *(4 items)*												
Cardiopulmonary Resuscitation (CPR) Bag/Mask	33.3	55.1	-	66.7	47.5	-	42.9	55.2	-	42.9	66.7	-
Oxygen Supply	6.7	6.9	-	73.3	47.5	-	7.1	17.2	-	64.3	62.5	-
IV Lines with Resuscitation Fluids	30.0	44.8	-	73.3	77.5	-	57.1	75.9	-	64.3	79.2	-
Anaphylaxis Medications (i.e., Antihistamine, Cortisone, and Adrenalin)	10.0	41.4	**+31.4** *	86.7	55.0	**−31.7** *	28.6	65.5	**+36.9** *	57.1	70.8	-
**Hygiene and Safety** *(4 items)*												
Adequate Lighting in Surgical Area	80.0	89.7	-	93.3	92.5	-	57.1	31.0	-	42.9	83.3	**+40.4** *
Adequate Ventilation in Surgical Area	90.0	93.1	-	86.7	72.5	-	64.3	93.1	**+28.8** *	42.9	87.5	**+44.6** *
Clean and Hygienic Surgical Area	93.3	100	-	86.7	55.0	**−31.7** *	57.1	72.4	-	42.9	91.7	**+48.8** *
Presence of Functional Adverse Events Monitoring System	93.3	96.5	-	60.0	22.5	**−37.5** *	92.9	96.6	-	100	95.8	-
**Service Package** *(5 items)*												
Medical Male Circumcision	100	100	-	100	100	-	100	100	-	100	100	-
VMMC Group Education	93.3	100	-	93.3	80.0	-	92.9	100	-	85.7	100	-
VMMC Individual Counseling	100	96.6	-	100	62.5	**−37.5** *	100	100	-	100	100	-
HIV Counseling and Testing	96.7	96.6	-	100	67.5	**−32.5** *	100	100	-	100	100	-
Referral Services	33.3	58.6	-	86.7	42.5	**−44.2** *	92.9	58.6	**−34.3** *	100	100	-

[A] Includes only percent of facilities with total score ( = 1 point). Items receiving a partial score ( = 0.5 point) at the facility were not counted as available.

* Differences from T_0_ to T_1_ reported at p<0.05 statistical significance.

### Quality of VMMC Surgeries

Table 3 summarizes the quality of observed VMMC surgeries. Pre-operative tasks were correctly done in 79.5% and 97.1% of observed surgeries in Kenya and Zimbabwe, respectively, during the initial year and were maintained during the second year: (Kenya: −8.5, 95% CI: −17.6, 0.6 and Zimbabwe: +2.9, 95% CI: 0.6, 5.2). Pre-operative tasks were omitted the most in Tanzania's observed surgeries with low scores during the initial (20.6%) and second (26.3%) year (+5.7, 95% CI: −3.4, 14.8). Pre-operative tasks were correctly done in half (52.1%) of observed surgeries in South Africa, although this significantly declined over time (−39.8, 95% CI: −47.7, −32.0).

**Table 3:** Summary of Quality of VMMC Surgical Care from Initial Year (2011) to Expansion Year (2012).

	Kenya			South Africa			Tanzania			Zimbabwe		
	2011 (T_0_)	2012 (T_1_)	Δ = T_1_- T_0_	2011 (T_0_)	2012 (T_1_)	Δ = T_1_- T_0_	2011 (T_0_)	2012 (T_1_)	Δ = T_1_- T_0_	2011 (T_0_)	2012 (T_1_)	Δ = T_1_- T_0_
Number of surgical observations	154	218	-	116	361	-	133	251	-	140	240	-
**Pre-Operative Assessment**												
Percent (%) surgical observations with 1 pre-operative task done	79.5	71.0	−8.5	52.1	12.3	**−39.8** *	20.6	26.3	+5.7	97.1	100	**+2.9** *
**Safe Operative Procedures**												
Percent (%) surgical observations with all 8 safe operative procedures done	4.1	4.7	+0.6	9.7	0.6	**−9.1** *	9.8	31.0	**+21.2** *	0	1.5	+1.5
Mean number (%) safe operative procedures done^A^	6.9 (86%)	6.8 (85%)	−0.1 (−1%)	6.6 (83%)	5.6 (70%)	**−1.0** * **(−13%** * **)**	6.3 (79%)	6.8 (85%)	**+0.5** * **(+6%** * **)**	6.5 (81%)	7.0 (87%)	**+0.5** * **(+6%)**
**Operative Techniques**												
Percent (%) surgical observations with all 13 operative techniques done	54.0	50.2	−3.8	66.9	33.7	**−33.2** *	66.4	36.7	**−29.7** *	39.3	94.1	**+54.8** *
Mean number (%) operative techniques done^A^	12.5 (96%)	12.4 (95%)	−0.1 (−1%)	12.4 (95%)	11.6 (89%)	**−0.8** * **(−6%** * **)**	12.5 (96%)	12.0 (92%)	**−0.5** * **(−4%** * **)**	11.5 (88%)	12.9 (99%)	**+1.4** * **(+11%** * **)**
**Post-Operative Assessment**												
Percent (%) surgical observations with all 7 post-operative tasks done	16.2	18.9	+2.7	2.6	0.6	−2.0	16.5	8.5	**−8.0** *	0	9.8	**+9.8** *
Mean number (%) post-operative tasks done^A^	4.4 (62%)	3.8 (54%)	**−0.6** * **(−8%** * **)**	4.0 (58%)	2.4 (35%)	**−1.6** * **(−23%** * **)**	4.5 (64%)	3.9 (55%)	**−0.6** * **(−9%** * **)**	4.9 (70%)	6.1 (87%)	**+1.2** * **(+17%** * **)**
**Composite Score**												
Percent (%) surgical observations with all 29 VMMC quality of care tasks done	1.4	1.4	-	0	0	-	0	1.7	+1.7	0	0	-
Mean number (%) VMMC quality of care tasks done^A^	24.6 (85%)	23.8 (82%)	**−0.8** * **(−3%** * **)**	23.5 (81%)	19.7 (68%)	**−3.8** * **(−13%** * **)**	23.6 (81%)	22.9 (79%)	**−0.7** * **(−2%** * **)**	23.8 (82%)	27.0 (93%)	**+3.2** * **(+11%** * **)**
**Quality Rating** B												
Excellent (score 27+)	27.2	19.3		15.0	0.8		13.5	12.4		10.7	92.7	
Good (score 24–26)	32.4	32.6	p = 0.11	30.8	7.8	p = 0.00	39.1	25.5	p = 0.05	52.9	4.4	p = 0.00
Fair (score 20–23)	33.1	39.0		29.2	33.8		36.1	45.0		29.3	0	
Poor (19 or less)	1.3	5.1		10.0	39.1		8.3	10.8		6.4	0	

[A] Includes only percent of surgical observations with total score ( = 1 point). Items receiving a partial score ( = 0.5 point) during observation were not counted as correctly done.

[B] Includes only facilities with no missing items. Thus, the sum of percentages does not equal zero.

* Denotes statistically significant at p<0.05.

Quality scores were highest during the operative period for surgical techniques. On average, 11.5 to 12.5 of 13 tasks were correctly done across countries. High scores were maintained during the follow-up year in Kenya (−0.1, 95% CI −0.3, 0.01) with significant improvements in Zimbabwe to 12.9 (+1.4, 95% CI 1.1, 1.7). Scores significantly decreased in South Africa to 11.6 (−0.8, 95% CI: −1.1,−0.5) and in Tanzania to 12.0 (−0.5, 95% CI: −0.7, −0.3). Scores for specific surgical tasks are shown in Table 4. Except for Zimbabwe, tying the surgical knot was consistently done incorrectly across countries, resulting in lower percentages of observed surgeries during expansion for which all 13 operative tasks were done correctly (Kenya 50.2%, South Africa 33.7%, Tanzania 36.7%, Zimbabwe 94.1%). Other incorrectly performed operative tasks were identification of the skin to be excised, administration of local anesthesia, and securing mattress sutures.

**Table 4:** Item-Level Quality of VMMC Surgical Care from Initial Year (2011) to Expansion Year (2012).

*Indicator:* Percent (%) of surgical observations with Total Score ( = 1 point) by Item^A^												
	Kenya			South Africa			Tanzania			Zimbabwe		
	2011 (T_0_)	2012 (T_1_)	Δ = T_1_- T_0_	2011 (T_0_)	2012 (T_1_)	Δ = T_1_- T_0_	2011 (T_0_)	2012 (T_1_)	Δ = T_1_- T_0_	2011 (T_0_)	2012 (T_1_)	Δ = T_1_- T_0_
Number of surgical observations	154	218	-	116	361	-	133	251	-	140	240	-
**Pre-Operative Assessment** *(1 item)*												
Performs preoperative assessment including targeted history and physical exam	79.5	70.6	-	51.7	12.2	**−39.5** *	20.3	26.3	-	97.1	100	**+2.9** *
**Safe Operative Procedures** *(8 items)*												
Uses sterile instruments and consumables	100	98.2	-	96.7	97.8	-	98.5	99.2	-	99.3	100	-
Uses sterile gloves	99.3	99.5	-	95.0	84.8	**−10.2** *	100	99.6	-	99.3	99.5	-
Hand washes or disinfects between clients	90.1	90.4	-	90.0	58.2	**−31.8** *	93.2	96.4	-	87.1	100	**+12.9** *
Maintains adequate sterile surgical field	100	95.4	**−4.6** *	85.0	76.7	-	57.1	79.3	**+22.2** *	80.7	100	**+19.3** *
Ensures protective eyewear worn by all surgical staff	5.3	6.4	-	10.8	5.3	-	25.6	55.4	**+29.8** *	0.7	2.5	-
Safely stores and disposes of medical waste	98.0	100	-	91.7	72.0	**−19.7** *	100	99.2	-	98.6	99.0	-
Correctly and hygienically processes instrument(s)	98.0	96.3	-	96.7	95.6	-	92.5	87.7	-	97.1	100	**+2.9** *
Disinfects surgical beds between patients	98.7	97.3	-	92.5	59.8	**−32.7** *	66.9	64.5	-	84.3	96.6	**+12.3** *
**Step-wise Operative Techniques** *(13 items)*												
Cleans surgical area with Chlorhexidine-based solution or Povidine Iodine	100	98.6	-	99.2	89.2	**−10.0** *	98.5	99.2	-	68.6	100	**+31.4** *
Identifies skin to be excised	100	95.9	**−4.1** *	97.5	70.1	**−27.4** *	93.2	84.1	**−9.1** *	91.4	100	**+8.6** *
Ensures no other penile parts are at risk of injury	100	100	-	96.7	96.7	-	99.3	98.4	-	95.7	99.5	**+3.8** *
Safely administers local anesthesia	98.0	89.9	**−8.1** *	89.2	70.4	**−18.8** *	97.0	90.0	**−7.0** *	89.3	100	**+10.7** *
Cautiously/gently removes foreskin	100	99.1	-	95.8	94.5	-	100	95.2	**−4.8** *	94.3	100	**+5.7** *
Uses electrocautery and/or ligating sutures	100	100	-	97.5	95.3	**−2.2** *	95.5	92.8	-	92.1	99.5	**+7.4** *
Correctly ties of surgical knot(s)	56.3	67.0	-	73.3	61.8	**−11.5** *	72.9	47.4	**−25.5** *	83.6	99.5	**+15.9** *
Aligns frenulum and secures mattress suture	100	98.6	-	98.3	87.5	**−10.8** *	100	99.2	-	90.0	100	**+10.0** *
Correctly aligns other quadrant sutures	99.3	99.5	-	94.2	96.4	-	100	99.2	-	92.1	100	**+7.9** *
Avoids placing deep sutures around the frenulum	100	100	-	98.3	96.7	-	100	99.2	-	93.6	94.6	-
Places interrupted sutures evenly	98.7	90.8	**−7.9** *	85.8	96.4	**+10.6** *	98.5	98.0	-	89.3	99.5	**+10.2** *
Ensures no significant bleeding present	100	100	-	97.5	94.2	-	100	99.2	-	85.0	99.5	**+14.5** *
Places secure dressing	98.7	98.7	-	98.3	96.4	-	98.5	99.6	-	88.6	100	**+11.4** *
**Post-Operative Assessment** *(7 items)*												
Waits to determine abnormality or allergic reaction	56.3	56.0	-	40.8	7.5	**−33.3** *	64.7	73.7	-	22.1	99.5	**+77.4** *
Reviews patient vital signs	44.4	33.9	**−10.5** *	66.7	58.2	-	48.9	42.6	-	0	9.8	**+9.8** *
Provides wound care and pain management advice	63.6	63.8	-	79.2	45.2	**−34.0** *	63.9	68.5	-	68.6	100	**+31.4** *
Encourages at least one patient follow-up visit	63.6	59.6	-	87.5	63.2	**−24.3** *	91.7	89.6	-	98.6	100	-
Provides emergency contact details to patient	96.0	91.3	-	66.7	35.2	**−31.5** *	82.0	68.5	**−13.5** *	98.6	99.5	-
Provides post-operative patient counseling	57.6	41.3	**−16.3** *	10.0	4.4	-	63.2	22.7	**−40.5** *	98.6	100	-
Reminds of 6-week post-operative abstinence	56.3	31.2	**−25.1** *	52.5	28.3	**−24.2** *	31.6	17.1	**−14.5** *	100	100	-

[A] Includes only percent of surgical observations with total score ( = 1 point). Items receiving a partial score ( = 0.5 point) during observation were not counted as correctly done.

* Differences from T_0_ to T_1_ reported at p<0.05 statistical significance.

While on average the majority of safe operative procedures were correctly done during the initial year (6.3 to 6.9 of the 8 minimum tasks), protective eyewear was often omitted in all 4 countries resulting in few surgeries where all 8 safety tasks were completed (Kenya: 4.1%, South Africa, 9.7%, Tanzania 9.8%, Zimbabwe 0%). Kenya maintained otherwise high safety scores (−0.1, 95% CI: −0.2, 0.04), with significant improvements in Tanzania (+0.5, 95% CI: 0.3, 0.7) and Zimbabwe (+0.5, 95% CI: 0.4, 0.6). Significantly fewer safety tasks were done in South Africa's expansion year as compared to the initial year (−1.0, 95% CI: −1.3, −0.7). Lower safety scores reflected lack of hand washing, not disinfecting hands between clients, or not maintaining sterile surgical fields.

Quality scores were lowest during the post-operative surgical period with significant decreases in three countries: Kenya, 4.4 to 3.8 out of 7 items (−0.6, 95% CI: −1.0, −0.2); South Africa, 4.0 to 2.4 (−1.6, 95% CI: −2.0, −1.3); and Tanzania, 4.5 to 3.9 (−0.6, 95% CI: −1.0, −0.2). Providers in Zimbabwe significantly increased the average number of post-operative tasks correctly done from 4.9 to 6.1 (+1.2, 95% CI: 1.1, 1.3). In the second year, only a small proportion of surgeries had all 7 post-operative tasks performed (Kenya 18.9%, South Africa 0.6%, Tanzania 8.5%, Zimbabwe 9.8%). Post-operative tasks usually omitted were monitoring patients for reactions and reviewing vital signs, as well as providing post-operative counseling and reminding patients of the post-operative abstinence period.

## Discussion

Scaling up VMMC programs is an important strategy in the prevention of HIV infection in sub-Saharan African countries. Previous VMMC quality assessments have often focused on reporting of adverse events. However, no research to-date has examined changes in facility preparedness and correct performance of VMMC procedures as countries scale-up the number of facilities. Given the potentially significant contribution of VMMC in decreasing HIV transmission, monitoring quality is critical in identifying and eliminating gaps in care during introductory and scale-up periods.

This study's comparative process evaluation demonstrated that high quality VMMC services can be provided during rapid scale-up in sub-Saharan African settings. Nearly all VMMC operative tasks observed in the study were performed correctly, with the exception of tying surgical knots. However, the evaluation revealed several areas of sub-optimal VMMC performance levels. Lower scores in infection control during surgery and pre- and post-operative tasks highlighted the need to reinforce provider training and supervision in these areas. Pre-operative examinations are necessary to assess eligibility for surgery and identify surgical contradictions which may lead to adverse events. Post-operative tasks are essential in prevention, identification, and management of complications. Reinforcement of counseling messages during the post-operative period on the importance of risk reduction and sexual abstinence during wound-healing is also crucial. Lower scores among these items could influence the overall effectiveness of VMMC given that transmission rates are directly affected by these behaviors.

When examining overall scores, there were considerable variations across the two years of program implementation. Results showed that high levels of service quality can be maintained at-scale and, in some cases, dramatically improved during the process of scale-up, as was the experience of Kenya and Zimbabwe, respectively. However, analyses also underscored the challenge of replicating initial successes at-scale in the case of South Africa, and the challenge of managing concurrent performance improvement and deterioration within the VMMC program in Tanzania. Some safety measures improved, while surgical technique and post-operative care quality declined. During the second year in both countries, less than a third of sampled sites had all requisite protocols to equip staff with readily accessible information. Equally as few sites had all necessary equipment and supplies for normal and emergency surgeries. The implications for expanding VMMC services in South Africa are further detailed in this supplement [18]. In all four countries, the absence of HIV post-exposure prophylaxis was more severe during the second year, and shortages at both time points of antibiotics and BLS equipment can hinder the management of severe adverse events.

Several lessons learned from this evaluation may inform future efforts to scale-up VMMC services in similar settings. One key finding is that while high quality VMMC programs can be achieved at-scale, the process of scaling-up does not guarantee compliance with all quality criteria. This was evidenced by the variability over time in facility preparedness and surgical care within countries. In some settings, quality measures declined over the two-year period, or the VMMC program was expanded in the second year with comparably low performance levels as observed during the first year. This evaluation emphasizes the need to routinely measure aspects of quality, and ensure that VMMC program expansion is linked with quality improvement initiatives.

Secondly, while the diversity of program experiences among countries precludes any definitive comparison, some insight may be drawn from the range of scale-up models which were used. All countries partnered with implementing agencies with variations in the implementation of fixed, mobile, and outreach sites. In addition, focused models provided the advantage of allocating resources to regions with greatest disparities in HIV prevalence and male circumcision, while national models benefited from population-wide implementation. Zimbabwe additionally delivered its VMMC program through a single implementing organization. There were also differences across countries in the volume of VMMC facilities added during the evaluation's two-year period. A final lesson emerging from this analysis is the recognition that challenges in commodity procurement and provider capacity may have reflected broader health systems strengthening needs that were not unique to VMMC. Careful attention will be needed to ensure that the urgency of scaling up VMMC does not bypass efforts to strengthen health systems or hinder quality of existing health services in facilities where VMMC is introduced.

### Limitations

The study's limitations deserve mention. VMMC facility preparedness was measured at the time of the study visit and does not reflect cumulative availability or adequacy of supplies relative to client load. Thus, use of point-in-time measures limited the evaluation's ability to examine seasonal trends in VMMC quality as the program expanded. Data were also collected during low volume service periods which may have confounded the study's observations, and the small number of facilities may have contributed to the wide confidence intervals of some measures. In addition, it is possible that in some instances the evaluation of VMMC surgeries was biased given that scores were based on the evaluator's interpretation. To minimize this, all observers were trained using a standardized observation guide. However, it was not possible to standardize ratings across countries which may have resulted in unequal scoring.

The evaluation's summary measures should likewise be interpreted carefully. Quality of VMMC services was examined from the perspective of the health system. Client perspectives of quality such as comfort, privacy, or communication adequacy are not reflected, and other systems-level quality metrics not measured in this study may have been informative. Furthermore, it can be argued that not all items were equally critical, and thus the analysis' equal weighting of items unduly magnified (or diluted) the clinical relevance of certain tasks. Although the development of differential weights was not feasible within the study's parameters, to reduce this possibility, we aimed to balance the interpretive weight of each score by including in the questionnaire only the minimally essential elements in each category. Thus, the range of scores is designed to reflect only items necessary to perform safe and effective circumcision. Finally, the results from this study are unique to the selected countries and may not be generalizable to other settings. This analysis was not designed to identify factors associated with differences in observed quality measures. Therefore, causal or comparative inferences cannot be drawn between countries or facilities.

### Conclusions

To our knowledge, this study is the first to examine facility preparedness and quality of VMMC services in contexts of scale-up in four African countries. Lapses in high performance and, in some cases, expansion of considerably deficient services emphasize the need to better integrate VMMC scale-up and quality improvement processes. Monitoring service quality, not just adverse event reporting, will be essential in realizing the full health impact of VMMC for HIV prevention in African settings.
